# Supplementary material for: End Sequence Analysis Toolkit (ESAT) expands the extractable information from single-cell RNA-seq data
Source: Genome Res. 2016 Oct;26(10):1397–410. doi: 10.1101/gr.207902.116 (PMC5052061; doi:10.1101/gr.207902.116)
Supplement: Supplemental Material [file supp_gr.207902.116_Supp_Materials.tar.gz › ESAT-esat_v0.1/doc/html/dir_fffd6c75032af08653295b9f32806ae0.html]

ESAT: java/broad/core/util Directory Reference


ESAT


- Main Page
- Related Pages
- Packages
- Classes
- Files

All Classes Namespaces Files Functions Variables Enumerator Pages

- java
- broad
- core
- util

util Directory Reference

Directory dependency graph for util:

|  |  |
| --- | --- |
| Files | |
| file | ChromosomeStringComparator.java |
|  | |
| file | CLUtil.java |
|  | |
| file | CollapseByIntersection.java |
|  | |
| file | GMTParser.java |
|  | |
| file | ParseGCTFile.java |
|  | |


---

Generated on Fri Aug 15 2014 15:11:16 for ESAT by  

 1.8.7
